# Implementation of the German Clinical Practice Guideline for Multimorbidity Using a Digital Tool in Primary Care: Pilot Cluster Randomized Clinical Trial

**DOI:** 10.2196/79767

**Published:** 2025-11-20

**Authors:** Julia Nothacker, Valentina Paucke, Susanne Lezius, Antonia Zapf, Dagmar Lühmann, Martin Scherer, Ingmar Schäfer

**Affiliations:** 1 Institute and Outpatients Clinic of General Practice and Primary Care University Medical Center Hamburg-Eppendorf Hamburg Germany; 2 Institute of Medical Biometry and Epidemiology University Medical Center Hamburg-Eppendorf Hamburg, null Germany

**Keywords:** multimorbidity, implementation, hospitalizations, clinical practice guidelines

## Abstract

**Background:**

Clinical practice guidelines (CPGs) summarize the best available evidence in a specific field. To improve patient-centered outcomes, guidelines have to be implemented, using, for example, information and communications technology. Although there are CPGs addressing multimorbidity, there is still a lack of studies investigating their implementation.

**Objective:**

This study aimed to evaluate whether the implementation of a CPG for multimorbidity using a digital tool is feasible and explore possible effects of this intervention.

**Methods:**

A pilot cluster randomized clinical trial based on telephone interviews was conducted from October 25, 2023, to September 8, 2024. Patients enrolled in any disease management program who were aged ≥65 years and had at least 2 additional chronic conditions were randomly selected from 20 general practitioner (GP) practices and contacted for informed consent. Each practice was randomized after baseline interviews of all participating patients in the practice were finished. The use of a web application facilitating collection and documentation of treatment-relevant data in accordance with the German CPG for multimorbidity was compared with treatment as usual. The primary outcome was time spent in hospital. As a secondary outcome, the number of patients with at least one hospital admission was calculated. Further secondary outcomes included outpatient health care use, quality of life, patient satisfaction, and quality of care. Feasibility assessment included examination of sample size, participation rate, and compliance with the study protocol. Outcome measures were analyzed using linear, logistic, and negative binomial regressions with random intercepts for practices.

**Results:**

Of 384 patients who were contacted, 123 (32%) agreed to participate, and 120 (31.3%, including 54/120, 45% in the intervention group and 66/120, 55% in the control group) completed baseline and follow-up assessments. Mean age was 75.4 (SD 6.6) years, and 51.7% (62/120) were women. The compliance rate, or the proportion of patients who were treated per protocol, was 89% (48/54). In our data, the incidence rate of hospital days was comparable in both groups (incidence rate ratio [IRR] 0.94, 95% CI 0.09-9.42; *P*=.96), but the odds of hospital admission were almost reduced by half in the intervention group (odds ratio 0.51, 95% CI 0.17-1.54; *P*=.23). Our data also suggest a higher incidence rate of GP contacts (IRR 1.13, 95% CI 0.83-1.53; *P*=.43) and lower incidence rate of contacts with outpatient specialists (IRR 0.79, 95% CI 0.54-1.15; *P*=.24) in the intervention group compared to usual care. Moreover, patients and GPs reported a better quality of care (mean difference 0.51, 95% CI −0.12 to 1.14; *P*=.12 and mean difference 1.19, 95% CI 0.13-2.25; *P*=.03, respectively) in the intervention group.

**Conclusions:**

Implementation of the CPG using a digital tool was feasible. Our data suggest that the probability of hospital admissions and contacts with outpatient specialists might be reduced and quality of care might be improved.

**Trial Registration:**

ClinicalTrials.gov NCT06061172; https://clinicaltrials.gov/study/NCT06061172

## Introduction

Clinical practice guidelines (CPGs) identify and summarize the best available evidence in a specific field [1]. To improve patient-centered outcomes, guidelines have to be implemented in regular health care. In contrast to development and dissemination, the implementation of guidelines is usually the responsibility of health care professionals [2]. There are various barriers to implementing guidelines, including time constraints and lack of resources [3,4]. One approach in optimizing guideline implementation is to provide structural changes by using information and communications technology [5,6]. A strength of this approach is facilitation of complex care [7] (eg, for patients with multimorbidity).

Multimorbidity is a common health problem in the primary care of older patients [8] that is associated with adverse outcomes such as decline in health-related quality of life [9] and increased risk of hospitalizations [10]. Patients with multimorbidity usually need tailored, person-centered care based on a prioritization of treatment goals [11]. In a recent systematic review, 20 guidelines specifically focusing on multimorbidity and polypharmacy were identified [12], but none have been implemented using a digital tool yet.

In Germany, the S3-level CPG for multimorbidity of the German Society of General Practitioners and Family Physicians does not focus on specific diseases but rather helps the general practitioner (GP) manage the patients’ individual situation [13]. A field test emphasized the high complexity of the treatment algorithm and the need for specific assessment tools [14]. Therefore, we developed a digital tool that facilitates the implementation of this guideline [15] and conducted a cluster randomized clinical pilot study that examined the feasibility of the full-scale evaluation of an intervention based on this tool and explored the possible effects of this intervention.

Specifically, the primary objective of this study was to assess whether participant enrollment, data acquisition, and the implementation of the intervention in the intended way are feasible. In addition, our pilot study aimed to consolidate our hypotheses by analyzing whether the intervention is capable of reducing the time patients spend in hospital and their outpatient health care use and whether it might improve quality of care, patients’ health-related quality of life, and patient satisfaction.

## Methods

### Study Oversight

Reporting followed the CONSORT (Consolidated Standards of Reporting Trials) extension for cluster randomized trials [16]. A project advisory board represented patients, caregivers, primary care physicians, self-governing bodies in the German health care system, the German professional society for primary care, and private companies developing digital therapeutics. The board informed the research team about the perspectives of health care providers and patients and provided guidance on how their interests and concerns can be considered in the design of our intervention and our pilot study.

### Ethical Considerations

The protocol for this pilot study was approved on June 27, 2022, and September 5, 2022, by the ethics committee of the Medical Association of Hamburg (2022-100786-BO-ff) and registered on September 24, 2023, in ClinicalTrials.gov (NCT06061172).

### Trial Design

We conducted a 2-group, parallel pilot cluster randomized clinical trial based on telephone interviews of older patients with multimorbidity and their GPs. On average, patient interviews lasted approximately 20 minutes, and GP interviews lasted approximately 10 minutes per patient. Answers were directly entered in deidentified form into Microsoft Excel spreadsheets. We conducted automated plausibility checks during data entry and retrospective evaluation of data quality before data preparation and analysis.

### Participants

A total of 20 GP practices in the German federal states of Bremen, Hamburg, Lower Saxony, and Schleswig-Holstein were recruited via mail as a convenience sample. We included practices who were known from other research projects and formerly unknown practices identified on the official websites of the regional associations of statutory health insurance physicians. GPs were eligible if (1) they were registered as statutory health insurance physicians in the metropolitan area of Hamburg or its rural surroundings; (2) they participated in a disease management program for asthma, coronary heart disease, diabetes mellitus, or chronic obstructive pulmonary disease; and (3) they could provide a list of patients from their practice who were aged ≥65 years and participated in such a program.

GPs created a numbered list of all patients who fulfilled these criteria. A member of the study team then assigned them random numbers using a mobile app. On the basis of these numbers, GPs selected patients from the list and screened them for multimorbidity and exclusion criteria until 20 eligible patients in each practice were identified. Multimorbidity was operationalized as 2 additional diagnoses considering the 46 most prevalent chronic diseases [17]. Exclusion criteria included having no capacity to consent, functional limitations precluding participation in the intervention (eg, loss of vision or no capacity to use a mobile device) or telephone interviews (eg, loss of hearing), limited German language skills, and participation in other trials during the pilot study.

Eligible patients received an invitation letter from their GP that included written patient information, 2 consent forms, and a prepaid return envelope. Patients who wanted to participate signed one consent form, gave their contact data, and returned both to the study center. Participating patients were then contacted and interviewed by the study team. In addition, attending GPs were interviewed about their patients and practice. Interviews were conducted at baseline and after the intervention period was finished (follow-up).

### Sample Size

As this was a pilot trial, we did not conduct a formal sample size calculation. For confirmation of feasibility, we wanted to include 10 practices for each treatment arm with, on average, 5 patients per practice. Allowing for a reasonable dropout, we aimed at recruiting 6 patients in each practice. In summary, our intended sample size was 20 practices with 120 patients altogether. This is suitable according to general recommendations for minimum sample sizes [18,19].

### Randomization

Each practice was randomized after the baseline interviews of all participating patients in the practice were finished. We used block randomization with computer-generated random numbers and variable block sizes stratified for region type (ie, rural and urban), which was generated by an independent statistician. The statistician received the practice number and region type via email but did not have access to any other patient or practice data. After randomization, the study team was informed about practice allocation via email and communicated this information to participating GPs via telephone. Patients were not actively informed about practice allocation but could not effectively be blinded.

### Intervention and Comparator

The intervention was based on the web application MultiTool (version 1.0.0; University Medical Center Hamburg-Eppendorf), which implements all recommendations of the German Society of General Practitioners and Family Physicians S3-level CPG for multimorbidity [13]. Guideline recommendations, specific functions, and other contents of the tool are described in detail in another publication [15].

The main functions of the tool were digital questionnaires in the domains of (1) patient preferences (ie, prioritization of treatment goals, control preferences, and involvement of other health care professionals), (2) social life (ie, social contacts, activities, and participation), (3) treatment situation (ie, medication adherence and treatment burden), and (4) health concerns (ie, pain, anxiety and depression, and other health concerns).

GPs could give patients access to the questionnaires via email, and patients could fill out the questionnaires at home or in the GP practice using any digital device with a browser and access to the internet. The tool also facilitated “brown bag” medication reviews [20] by providing a standardized invitation letter clarifying which drugs patients should bring to the consultations and a guide on how the reviews should be structured by the GPs. Results from discussions between GPs and patients could be documented within the tool.

GPs in the intervention group received access to MultiTool, a short training on the digital tool and the intervention, a written manual, and contact data for telephone support. Approximately 3 months after randomization, the practices allocated to the intervention group were contacted via telephone by the study team to monitor the implementation of the intervention. During this call, possible adverse events were assessed by asking GPs in unstandardized form whether any undesirable or harmful event had occurred during the observation period, which may or may not be related to the intervention [21].

GPs in the intervention group had to use MultiTool for every patient participating in the trial at least once in every 3-month accounting period. They were further instructed, first, to use the questionnaire for prioritization of treatment goals and at least one questionnaire in each of the domains of social life, treatment situation, and health concerns. Second, the clinical consequences of the patients’ answers had to be discussed between GPs and patients, and third, at least one medication review during the trial was obligatory. Compliance with the study protocol was reported by GPs and defined as carrying out these 3 instructions with their patients.

GPs in the control group had no access to MultiTool and provided care as usual. Exposure time in both groups was 6 months starting from the randomization of the practice.

### Clinical Outcomes and Other Variables

The primary outcome measure was time spent in hospital during the preceding 6 months. It was assessed in patient interviews by asking the patients how often they had been in hospital and how many days each hospital stay had lasted. The total time in hospital was calculated by adding the duration of all stays. As a secondary outcome, the number of patients with at least one hospital admission during the previous 6 months was also calculated.

Further secondary outcome measures included the number of contacts with the GP, outpatient specialists, and home care services during the preceding 6 months. Self-rated health was assessed on a scale from 0 to 100. Health-related quality of life was measured using the EQ-5D-5L, which comprises the domains of mobility, self-care, usual activities, pain or discomfort, and anxiety or depression [22], and the corresponding German value set, which assumes a value of 1 for perfect health and subtracts values between 0.026 and 0.612 for limitations in each of the 5 domains [23]. Patient satisfaction with clinical performance (17 items) and organization of care (6 items) were assessed on a 5-point Likert scale (0=bad; 4=excellent) using the European Task Force on Patient Evaluation of General Practice instrument [24]. For both subscales, a summary score was calculated by adding the values of each nonmissing item and dividing by the number of available answers. All these data were collected in patient interviews. In addition, quality of care was assessed using patient-reported (7 items) and GP-reported (12 items) summary scores of validated quality indicators for the primary care of patients with multimorbidity [25]. These scores were calculated by adding 1 point for each fulfilled indicator.

Patient interviews also included data on age, sex, marital status, living arrangement, educational level, and migration background. The educational level was coded using the Comparative Analysis of Social Mobility in Industrial Nations classification of tertiary, secondary, and primary education and lower [26]. Migration background was defined by the country of birth of the study participants and their parents. Moreover, depressiveness was assessed in patient interviews using a summary score of the 15 items of the Geriatric Depression Scale [27], which gives 1 point for each positive answer. Additionally, the number of chronic conditions was collected in GP interviews.

### Feasibility Assessment

To analyze the feasibility of recruitment of practices and patients, sample size and participation rate were analyzed. A priori, we expected that 400 eligible individuals could be identified and contacted and that 120 participants could be enrolled, which corresponds to an anticipated participation rate of 30%. To assess the quality of data acquisition, we analyzed the number of missing values for each variable. To examine the implementation of the intervention, we analyzed how many participants in each practice were compliant with the study protocol.

### Statistical Analyses

Descriptive comparisons between the intervention and control groups were reported using means and SDs for continuous data and numbers and percentages for categorical data. Potential differences between patients in the intervention and control groups regarding their baseline characteristics and the primary and secondary outcomes were assessed using 2-tailed *t* tests and chi-square tests. Distinct baseline imbalances were defined by *P* values of <.05. Rates of change were calculated using the following formula:



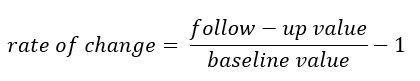



**(1)**

According to their type, primary and secondary outcomes were analyzed using multilevel mixed-effects negative binomial, logistic, or linear regression, and the respective effect measures with corresponding 95% CIs were reported. All statistical models were adjusted for random effects at the level of GP practices, and negative binomial and linear regression models were also adjusted at the level of patients within practices. The secondary outcome of “proportion of patients who stayed in hospitals” was compared at follow-up and controlled for the number of hospital stays at baseline. Intraclass correlation coefficients were reported to quantify the correlation among observation within the clusters. For all analyses, the available dataset was used.

The capability of our intervention to influence outcomes was determined by the effect sizes of these analyses. As the power of our pilot study was not adequate, hypotheses were consolidated solely based on regression coefficients. In addition, the reported *P* values are to be understood as descriptive measures and not used for confirmatory conclusions. All statistical analyses were conducted using Stata (version 15.1; StataCorp) [28].

## Results

### Participants

**Figure 1:**
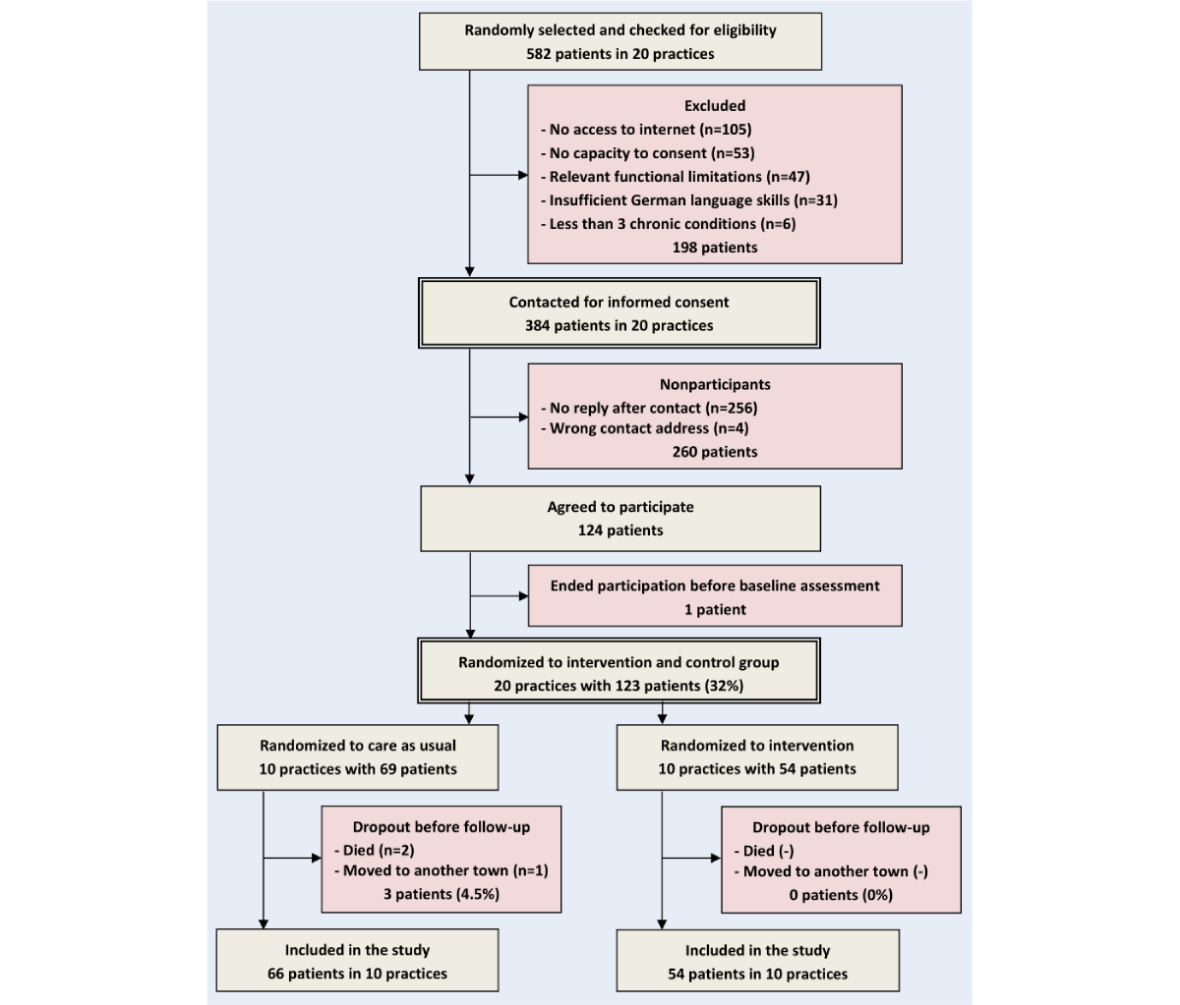
Sampling and response rates.

The participant flow can be found in Figure 1. A total of 582 patients from 20 practices were screened for eligibility, and 384 (66%) were contacted by their GPs for informed consent. Baseline interviews were conducted between October 25, 2023, and January 31, 2024. Subsequently, 20 practices with 32% (123/384) of the contacted patients were randomized to the intervention and control groups. A total of 1.6% (2/123) of the patients died during the observation period, and 0.8% (1/123) moved to another town, both in the control group. The dropout rate was 2.4% (3/123) altogether. The final sample size was 66 patients in the control group and 54 patients in the intervention group. Follow-up interviews were conducted between June 3, 2024, and September 8, 2024.

### Clinical Outcomes and Other Variables

On average, GPs in the control group were aged 52.0 (SD 8.4) years, and GPs in the intervention group were aged 54.1 (SD 8.4) years. In both groups, 60% (6/10) of the GPs were men, and 40% (4/10) were women. Both groups had a comparable mean number of GPs working in the practices (3.0, SD 1.5 in the intervention group vs 3.0, SD 2.0 in the control group) and a comparable number of treated patients per 3-month accounting period (mean 2300, SD 558 in the intervention group vs mean 2560, SD 1285 in the control group). The sociodemographic data of patients at baseline are reported in Table 1. There were no distinct baseline imbalances related to age (mean 75.8, SD 6.5 vs mean 74.9, SD 6.8 years; *P*=.48), sex (35/66, 53% men in the intervention group vs 23/54, 43% men in the control group; *P*=.26), or other sociodemographic data, but the mean number of chronic conditions in the control group was distinctly higher than that in the intervention group (10.4, SD 4.8 vs 8.8, SD 3.1, respectively; *P*=.03). The summary score on the Geriatric Depression Scale was comparable between both groups (mean 6.0, SD 1.5 vs mean 6.1, SD 1.7; *P*=.59).

There were no distinct imbalances in primary and secondary outcome measures at baseline between the control and intervention groups (Table 2). However, in the control group, fewer days in hospital during the 6 months before the baseline assessment were reported than in the intervention group (mean 0.9, SD 3.5 vs mean 2.4, SD 6.9, respectively; *P*=.14), and the probability of at least one hospital admission was also lower (11/66, 17% in the intervention group vs 12/54, 22% in the control group; *P*=.44). At follow-up, the probability of at least one hospital admission increased in the control group and decreased in the intervention group (13/66, 20% vs 8/54, 15%, respectively).

Home care services were only used by 6.7% (8/120) of the participants at baseline and 4.2% (5/120) of the participants at follow-up, and therefore, the effect of the intervention on the number of contacts with these services could not be analyzed. There was one missing value (1/66, 2%) for GP-reported quality of care in the control group and another missing value (1/54, 2%) for health-related quality of life scores in the intervention group. All other variables did not have any missing values.

**Table 1:** Sociodemographic data of the patients at baseline.

Characteristics			Control group (n=66)		Intervention group (n=54)		*P* value
Age (y), mean (SD)			75.8 (6.5)		74.9 (6.8)		.48
Sex (male), n (%)			35 (53)		23 (43)		.26
**Marital status, n (%)**							.38
	Married	48 (73)		31 (57)			
	Widowed	12 (18)		15 (28)			
	Divorced	3 (5)		4 (7)			
	Single	3 (5)		4 (7)			
Living arrangement (alone), n (%)			13 (20)		19 (35)		.06
**Educational level, n (%)**							.49
	Primary or lower	42 (64)		29 (54)			
	Secondary	16 (24)		15 (28)			
	Tertiary	8 (12)		10 (19)			
**Migration status, n (%)**							.64
	Patient and parents born in Germany	49 (74)		38 (70)			
	Patient or at least one parent born abroad	17 (26)		16 (30)			

**Table 2:** Differences in primary and secondary outcomes at baseline and follow-up between the control and intervention groups.

				Control group		Intervention group		*P* value
**Primary outcome**								
	**Days in hospital (previous 6 mo)**							.14
		Baseline, mean (SD)	0.9 (3.5)		2.4 (6.9)			
		Follow-up, mean (SD)	1.3 (4.1)		3.1 (9.8)			
		Change, %	+38		+31			
**Secondary outcomes**								
	**Number of contacts with GP^a^** **(previous 6 mo)**							.37
		Baseline, mean (SD)	3.7 (2.7)		3.3 (2.7)			
		Follow-up, mean (SD)	2.8 (2.1)		2.7 (1.8)			
		Change, %	−25		−16			
	**Number of contacts with outpatient specialists (previous 6 mo)**							.35
		Baseline, mean (SD)	1.6 (1.5)		1.9 (2.1)			
		Follow-up, mean (SD)	2.0 (2.2)		1.9 (1.7)			
		Change, %	+24		−2			
	**GP-reported quality of care (previous 6 mo, 0-12)**							.92
		Baseline, mean (SD)	8.0 (2.6)		7.9 (2.8)			
		Follow-up, mean (SD)	9.0 (3.0)		10 (1.6)			
		Change, %	+13		+28			
	**Patient-reported quality of care (previous 6 mo, 0-7)**							.17
		Baseline, mean (SD)	3.0 (2.2)		3.6 (1.9)			
		Follow-up, mean (SD)	2.9 (2.1)		3.9 (2.2)			
		Change, %	−5		+10			
	**Self-rated health—EQ-5D-5L visual analog scale (0-100)**							.94
		Baseline, mean (SD)	72 (18)		72 (19)			
		Follow-up, mean (SD)	73 (21)		75 (17)			
		Change, %	+1		+4			
	**Health-related quality of life—EQ-5D-5L German value set (–0.661 to 1)**							.14
		Baseline, mean (SD)	0.8 (0.2)		0.8 (0.2)			
		Follow-up, mean (SD)	0.8 (0.2)		0.9 (0.2)			
		Change, %	+3		+1			
	**Patient satisfaction—EUROPEP^b^** **clinical performance subscale (0-4)**							.11
		Baseline, mean (SD)	3.2 (0.7)		3.4 (0.6)			
		Follow-up, mean (SD)	3.1 (0.9)		3.4 (0.6)			
		Change, %	−4		−2			
	**Patient satisfaction—EUROPEP organization of care subscale (0-4)**							.71
		Baseline, mean (SD)	3.2 (0.7)		3.1 (0.7)			
		Follow-up, mean (SD)	3.1 (0.7)		3.1 (0.7)			
		Change, %	−3		−3			

^a^GP: general practitioner.

^b^EUROPEP: European Task Force on Patient Evaluation of General Practice.

### Feasibility Assessment

The intervention started in the first practice on November 27, 2023, and ended in the last practice on August 1, 2024. Table 3 provides an overview of compliance with the study protocol in each practice. In 70% (7/10) of the practices, all elements of the intervention were implemented per protocol with every participating patient, and in 30% (3/10) of the practices, the protocol was only partially followed. In summary, 98% (53/54) of the patients conducted assessments, 89% (48/54) participated in the intended discussions about the assessment results, and 98% (53/54) received a structured medication review. The compliance rate of patients who were treated per protocol was 89% (48/54). During the intervention, no adverse effects were reported by the GPs.

**Table 3:** Compliance with the study protocol by practice.

	Conducted assessments, n (%)	Discussion of assessment results, n (%)	Structured medication review, n (%)
Practice 1 (n=8 patients)	8 (100)	8 (100)	8 (100)
Practice 2 (n=5 patients)	4 (80)	4 (80)	4 (80)
Practice 3 (n=6 patients)	6 (100)	6 (100)	6 (100)
Practice 4 (n=2 patients)	2 (100)	0 (0)	2 (100)
Practice 5 (n=2 patients)	2 (100)	2 (100)	2 (100)
Practice 6 (n=5 patients)	5 (100)	5 (100)	5 (100)
Practice 7 (n=4 patients)	4 (100)	4 (100)	4 (100)
Practice 8 (n=10 patients)	10 (100)	10 (100)	10 (100)
Practice 9 (n=8 patients)	8 (100)	8 (100)	8 (100)
Practice 10 (n=4 patients)	4 (100)	1 (25)	4 (100)

### Statistical Analyses

Associations between the intervention and primary and secondary outcome measures are shown in Table 4. In the analysis of the primary end point, in both groups, a comparable incidence rate of hospital days was detected (incidence rate ratio [IRR] 0.94, 95% CI 0.09-9.42; *P*=.96). In contrast, the alternative operationalization of the primary end point indicated almost half the odds of hospitalization in the intervention group (odds ratio 0.51, 95% CI 0.17-1.54; *P*=.23). Our data also suggest a higher incidence rate of GP contacts in the intervention group (IRR 1.13, 95% CI 0.83-1.53; *P*=.43) and a lower incidence rate of contacts with outpatient specialists (IRR 0.79, 95% CI 0.54-1.15; *P*=.24) compared to care as usual. Patients reported a higher quality of care in the intervention group (mean difference 0.51, 95% CI −0.12 to 1.14; *P*=.12), and the intervention was also associated with higher GP-reported quality of care (mean difference 1.19, 95% CI 0.13-2.25; *P*=.03). We observed no relevant association with the other secondary outcome measures.

**Table 4:** Ratios and differences between the control and intervention groups in the primary and secondary outcomes.

Outcome	Measure (95% CI)	*P* value	ICC_1_^a^	ICC_2_^b^
Days in hospital (primary): incidence rate ratio^c^	0.94 (0.09 to 9.42)	.96	—^d^	—^d^
At least one hospital admission (secondary): odds ratio^e^	0.51 (0.17 to 1.54)	.23	0.013	—^d^
Number of contacts with GP^f^ (secondary): incidence rate ratio^c^	1.13 (0.83 to 1.53)	.43	—^d^	—^d^
Number of contacts with outpatient specialists (secondary): incidence rate ratio^c^	0.79 (0.54 to 1.15)	.24	—^d^	—^d^
GP-reported quality of care (secondary): mean difference^g^	1.19 (0.13 to 2.25)	.03	0.223	0.348
Patient-reported quality of care (secondary): mean difference^g^	0.51 (−0.12 to 1.14)	.12	0.091	0.649
Self-rated health—EQ-5D-5L visual analog scale (secondary): mean difference^g^	1.98 (−4.36 to 8.33)	.54	0.017	0.553
Health-related quality of life—EQ-5D-5L German value set (secondary): mean difference^g^	−0.02 (−0.08 to 0.05)	.58	0.002	0.661
Patient satisfaction—EUROPEP^h^ clinical performance subscale (secondary): mean difference^g^	0.07 (−0.12 to 0.27)	.45	0.066	0.703
Patient satisfaction—EUROPEP organization of care subscale (secondary): mean difference^g^	0.04 (−0.18 to 0.26)	.70	0.132	0.626

^a^ICC_1_: intraclass correlation coefficient at the practice level.

^b^ICC_2_: intraclass correlation coefficient at the patient in practice level.

^c^Analyzed using multilevel mixed-effects negative binomial regression.

^d^Not applicable.

^e^Analyzed using multilevel mixed-effects logistic regression.

^f^GP: general practitioner.

^g^Analyzed using multilevel mixed-effects linear regression.

^h^EUROPEP: European Task Force on Patient Evaluation of General Practice.

## Discussion

### Principal Findings

In this pilot study, we were able to enroll the intended number of patients and collect almost all the intended data, and for 89% (48/54) of the patients, the intervention was implemented per protocol. Our results suggest that fewer hospital admissions, a better quality of care [25], and a reduced number of contacts with outpatient specialists could be effects of the intervention. Similarly, an increase in the incidence rate of contacts with the GP is a possible result of the intervention.

### Comparison With the Literature

Guideline implementation using only written and oral dissemination is known to be ineffective [29]. Instead, proactive strategies tailored to each setting and target group are recommended to improve the management of chronic conditions [30,31]. However, to date, many implementation strategies have not been researched sufficiently [32], and few digital health interventions for the management of chronic diseases have demonstrated benefits in patient care [33].

For example, a clinical decision support system comprising recommendations from several single-disease guidelines led to a reduction in cardiovascular risk factors in patients with diabetes and a reduced risk of hospitalization [34]. Although a scoping review identified 3 other studies based on clinical guidelines [35], one did not report any benefits of the intervention [36], and the others did not assess any clinical outcomes [37,38]. Clinical results of an ongoing study using evidence-based, computerized decision-making for older patients with multimorbidity are still pending [39-41].

The high level of complexity (eg, due to heterogeneity of the population [17,42], fragmented care [43-45], and polypharmacy [8,46]) leads to difficulties in testing interventions designed to improve health outcomes for patients with multimorbidity [47]. A Cochrane systematic review comprising 17 randomized clinical trials [47] found little differences in clinical outcomes (eg, small improvements in patient-reported outcomes and medication adherence). Most of the studies included in the aforementioned Cochrane review and other reviews [48,49] did not find significant differences in hospitalizations.

### Implications

Our pilot study demonstrated that implementation of a CPG for multimorbidity in GP practices using a digital tool is feasible, and our data suggest that patient-oriented outcomes can possibly be improved. Therefore, policymakers and clinicians should facilitate digital implementation of existing guidelines for multimorbidity, but due to the lack of robust data on their effectiveness, these approaches need to be thoroughly evaluated.

With regard to our target group, other studies have pointed to possible barriers, including functional impairment and limited adherence [50-52]. In our study, 98% (53/54) of the older patients with multiple chronic conditions shared their data using the digital tool and participated in digitally assisted medication reviews. One facilitator in our study might be that the digital tool was developed and customized with participation of the target groups [15]. Therefore, similar studies should also consider participatory approaches.

Hospitalization is associated with multimorbidity [10], and it is a highly relevant outcome for this patient group [53]. However, there are various ways to operationalize hospitalization, and few interventions have reduced the number of hospitalizations of patients with multimorbidity [49]. In this context, our data suggest that the probability of at least one hospitalization might be a better operationalization than the number of hospital days.

Another implication for research is that health care use should be monitored when similar interventions are conducted. In line with other studies strengthening primary care [54,55], our data suggest that, on the one hand, the number of contacts with outpatient specialists could be reduced but, on the other hand, the number of contacts with GPs might increase due to this intervention.

### Strengths and Limitations

The strengths of this study include the experimental design with pretest-posttest comparison, 2 treatment groups, cluster randomization, and prospective study registration, which all contribute to the robustness of our analyses. The sample size was based on recommendations from the literature. Interviewers received standardized training in advance, and data quality was further improved through numerous automated and retrospective plausibility checks. In-person interviews by telephone were chosen to increase the response rate and ensure that study participants were able to ask questions during the interviews, which made wrong answers due to misunderstandings less likely. With 1078 of 1080 values in outcome variables being available, we only had 0.19% (2/1080) of missing values in our data. Multilevel mixed-effects regression analyses considered similarities between patients and between treatment approaches at the practice level. In addition, participation of the target groups in the development of the intervention [15] and a project advisory board representing patients and the public improved the clinical relevance and applicability of our intervention.

However, due to the pilot study design and missing adjustment for multiplicity, the results of our analyses have to be interpreted as only exploratory and not confirmatory. Other limitations of this study include different sample sizes in the intervention and control group, which reduce the power of the study. We also found mild but mostly indistinct imbalances at baseline [56]. Therefore, we will include the number of recruited patients as an additional stratum for randomization, and sensitivity analyses [57] will be adjusted for possible confounding. However, as the full-scale evaluation will have a sample size 5 to 6 times higher, we expect that differences between both groups will generally be less pronounced.

Another limitation is the compliance rate of 89% (48/54) of patients being treated per protocol [58], which is still acceptable in comparison with those of other studies [59,60]. In the evaluation study, we will improve compliance with the trial protocol through a stricter monitoring of practices. In addition, we will equip intervention practices with tablet computers to facilitate digital assessments for patients without their own mobile devices.

To optimize outcome measurement in the evaluation study, we tested 2 operationalizations of the primary outcome, one of which was not defined in the registered protocol. Therefore, results regarding this measure should be interpreted with caution. Moreover, outcomes were assessed via self-reports, which are prone to social desirability bias, recall problems, and errors. In contrast to this pilot study, it is also planned to blind assessors in the full-scale evaluation.

### Conclusions

This pilot study demonstrated that participant enrollment and data acquisition for evaluation and implementation of the intervention were feasible. Our data suggest that the probability of hospital admissions and contacts with outpatient specialists might be reduced and quality of care might be improved by our intervention. A full-scale cluster randomized clinical trial is now warranted to confirm these effects.

## Appendix

Multimedia Appendix 1 CONSORT-EHEALTH checklist (V 1.6.x).

## Abbreviations

CONSORT: Consolidated Standards of Reporting Trials
CPG: clinical practice guideline
GP: general practitioner
IRR: incidence rate ratio
